# The Indian Famine

**Published:** 1900-02-24

**Authors:** 


### THE INDIAN FAMINE.

### HELP REQUIRED.

The famine in India continues to cause a terrible amount of distress, and help is much needed. In spite of the large sums devoted to the sufferers from the war the Famine Fund at the Mansion House is rapidly growing. Help in kind is also much appreciated, and the following information is issued by the India Office for the guidance of intending donors:—

Memorandum.

Donors of parcels of clothing, &c., who desire that their gifts be transmitted to India by the Director-General of Stores for India, are recommended to adopt the following procedure, otherwise delay may occur in the despatch of the gifts to that country.

1. Each parcel must be clearly addressed—
The Secretary to the Government of Bombay,
Famine Department,
Poona,
and bear a label stating the name and address of the donor and the estimated value (for Customs) of the contents.

2. All parcels to be sent, carriage paid, to the India Store Depôt (Parcel Post Branch), Belvedere Road, Lambeth, London.

3. Advice of each consignment is to be sent to the Director-General of Stores, India Office, Whitehall, London, by whom its receipt will be duly acknowledged.

(Signed) E. Grant Burls,
Director-General of Stores for India.

February, 1900.

Description of Clothing Required.

A Hindu woman actually needs but two garments—a skirt and a chadar, or veil. To make the skirt, take 5 yards of print gingham, flannelette, or any closely-woven cotton goods, and sew the two ends together. Along one edge of 30-inch print add 8 inches of Turkey red, dark blue, or any contrasting colour of cotton goods. Turn down a hem 1¼ inch wide and run in a stout drawing string. To the bottom add a 3-inch bias strip of the same material as used at the top. Turn up a 2-inch hem, leaving 1 inch of the bias strip as additional length. The veil should be a plain colour, of white, red, dark blue, or any of the bright colours as produced in butter cloth. It may be of any sort of thick or thin cotton material, stout unbleached muslin, butter cloth, remnants of dress lining, old muslin or lace curtains, anything of the kind, only it must be 3 yards long and 1¼ yards wide.

Thousands of hard-working men would wear around their shoulders such a chadar of stout white muslin in lieu of jacket, and it would serve them as wrapping at night.

Native Christian women and little girls wear skirts with plain hems of the skirt materials at top and bottom, and many such could be made. Bundles of remnants should be added to the parcels for making jackets and trousers or divided skirts for Mahommedan women, which could be better done in India.

Cast-off clothing, as hats, bonnets, dress bodices, jackets, and men's wearing apparel are entirely unsuitable for distribution in India. Old skirts which could be made over for children would be useful. All manner of sheets, blankets, and quilts are greatly needed. When these are made of new material they should be 3 yards in length and from 1¼ to 1½ yards in width, as such will answer for day as well as night wear.

